# Supplementary material for: Evolutionary Divergence of Phosphorylation to Regulate Interactive Protein Networks in Lower and Higher Species
Source: Int J Mol Sci. 2022 Nov 20;23(22):14429. doi: 10.3390/ijms232214429 (PMC9697241; doi:10.3390/ijms232214429)
Supplement: Supplementary file 1 [file ijms-23-14429-s001.zip › supplementary_figures.pdf]

Supplementary figures

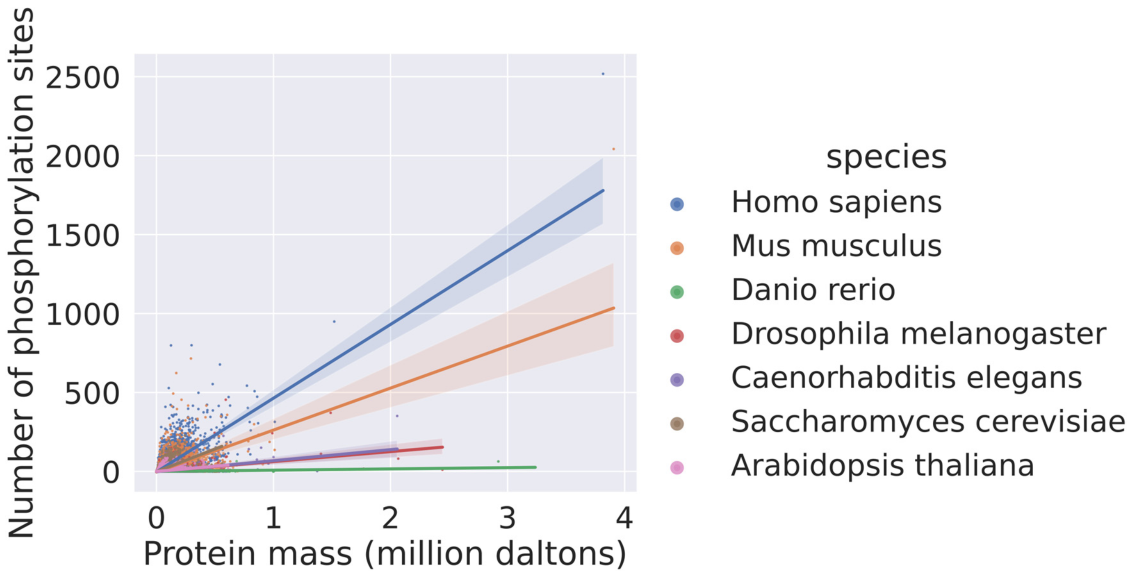

Figure S1 Plot of the relationship between masses and the number of phosphorylation sites for each phosphorylated protein

Regressions lines are drawn with a confidence interval of 0.95 (highlighted using translucent bands around the regression line).

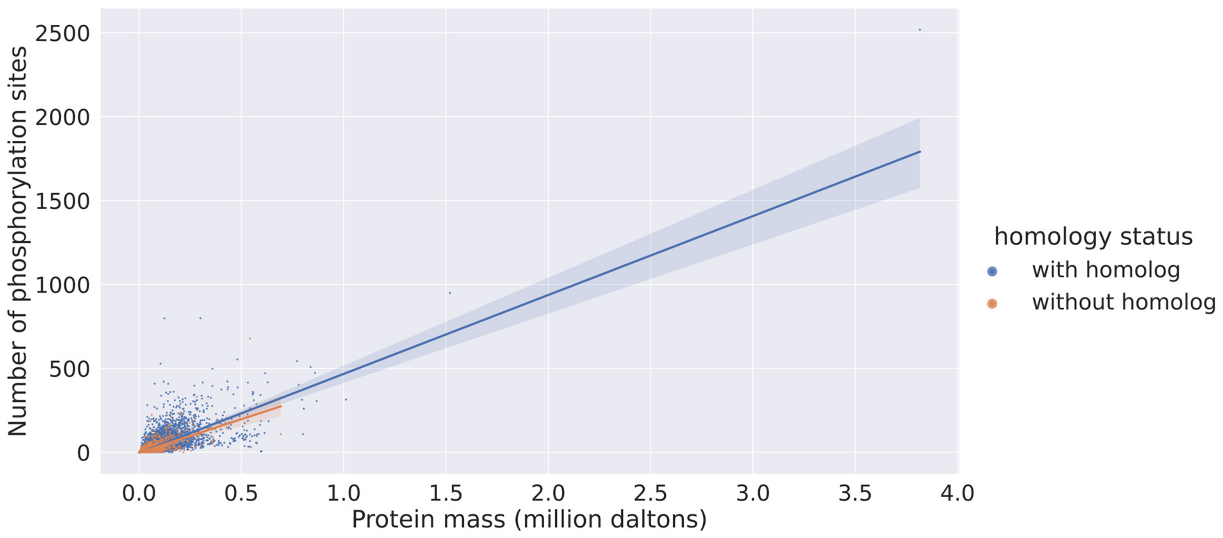

Figure S2 Plot of the relationship between masses and the number of phosphorylation sites for each phosphorylated protein in Homo sapiens

Data in blue are for proteins that have at least one homolog in another species while data in orange are for proteins without homolog. Regressions lines are drawn with a confidence interval of 0.95 (highlighted using translucent bands around the regression line).

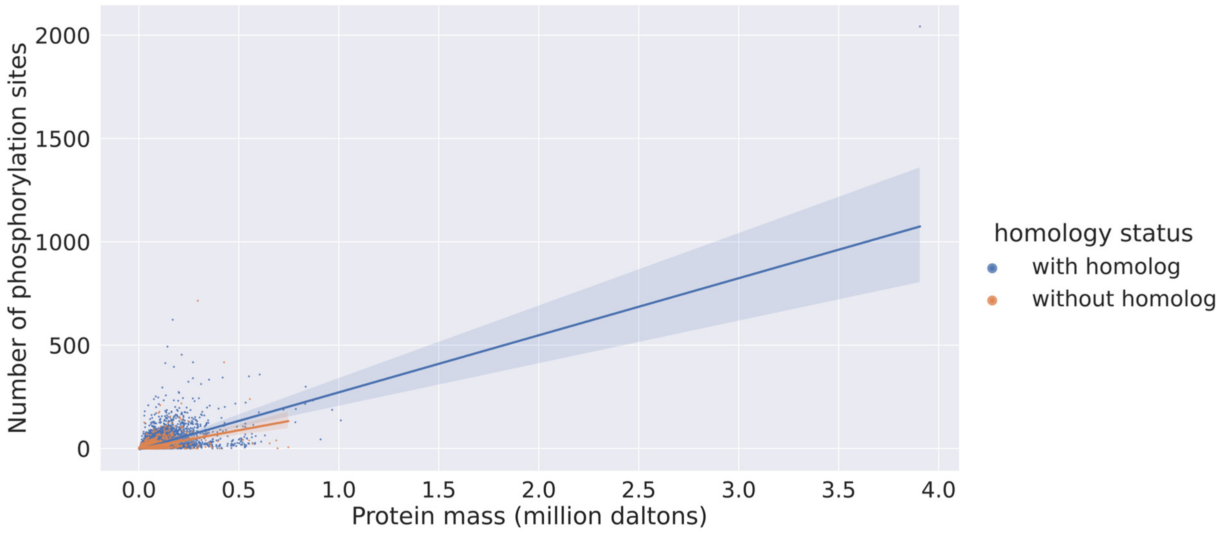

Figure S3 Plot of the relationship between masses and the number of phosphorylation sites for each phosphorylated protein in Mus musculus
Data in blue are for proteins that have at least one homolog in another species while data in orange are for proteins without homolog. Regressions lines are drawn with a confidence interval of 0.95 (highlighted using translucent bands around the regression line).

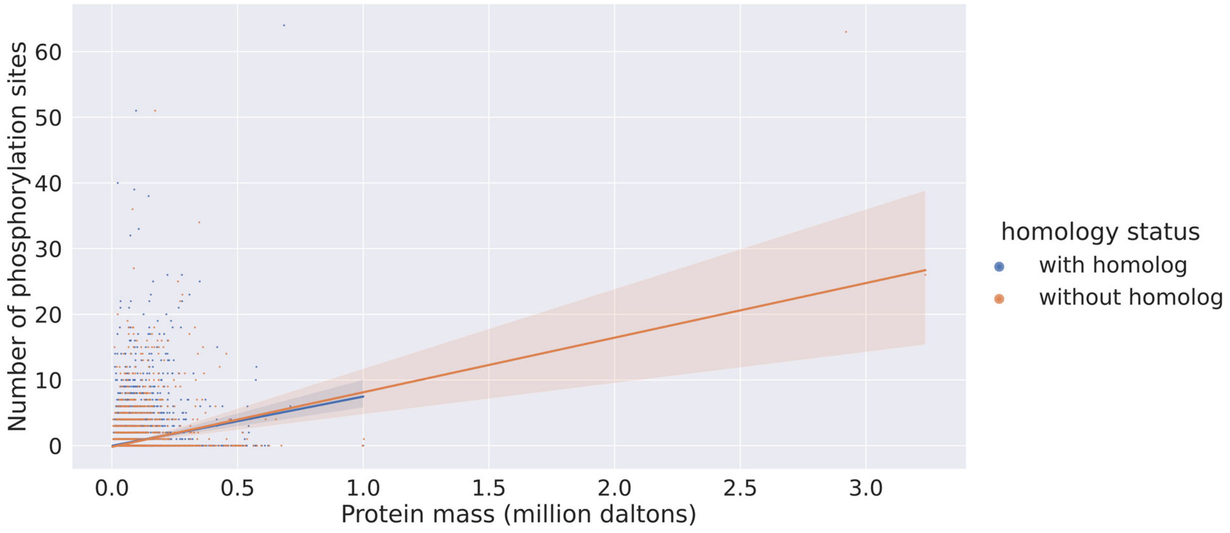

Figure S4 Plot of the relationship between masses and the number of phosphorylation sites for each phosphorylated protein in Danio rerio
Data in blue are for proteins that have at least one homolog in another species while data in orange are for proteins without homolog. Regressions lines are drawn with a confidence interval of 0.95 (highlighted using translucent bands around the regression line).

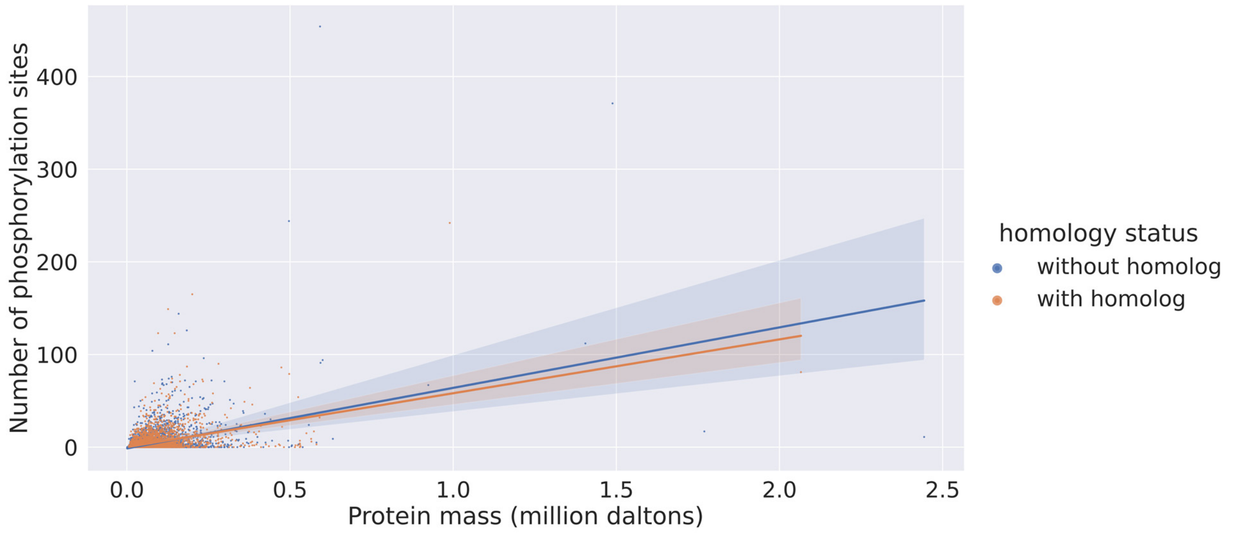

Figure S5 Plot of the relationship between masses and the number of phosphorylation sites for each phosphorylated protein in Drosophila melanogaster
Data in blue are for proteins that have at least one homolog in another species while data in orange are for proteins without homolog. Regressions lines are drawn with a confidence interval of 0.95

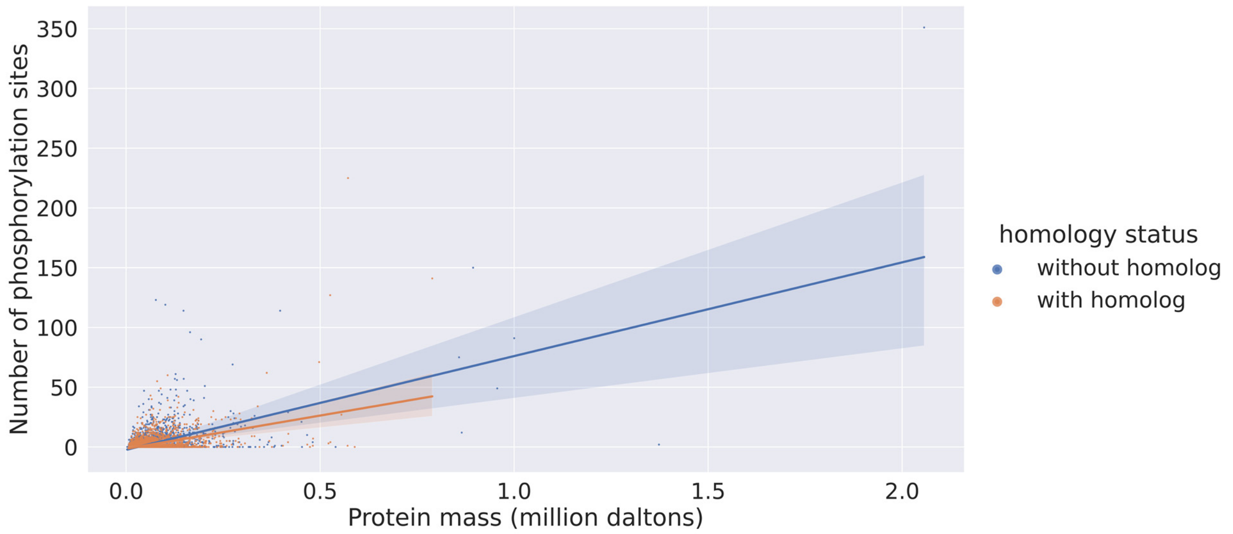

(highlighted using translucent bands around the regression line).

Figure S6 Plot of the relationship between masses and the number of phosphorylation sites for each phosphorylated protein in Caenorhabditis elegans
Data in blue are for proteins that have at least one homolog in another species while data in orange are for proteins without homolog. Regressions lines are drawn with a confidence interval of 0.95 (highlighted using translucent bands around the regression line).

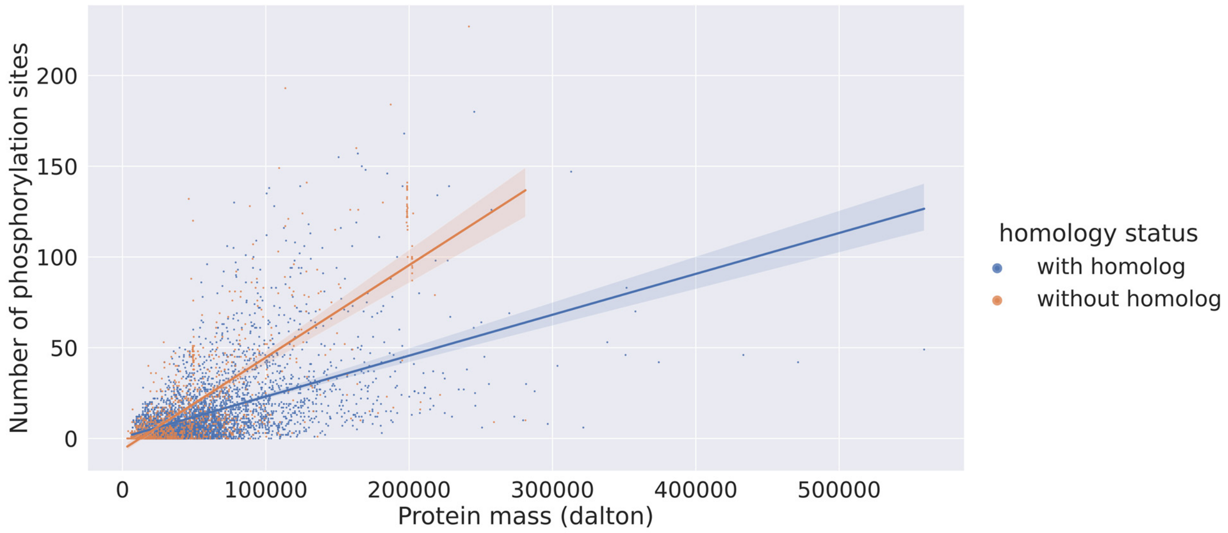

Figure S7 Plot of the relationship between masses and the number of phosphorylation sites for each phosphorylated protein in Saccharomyces cerevisiae

Data in blue are for proteins that have at least one homolog in another species while data in orange are for proteins without homolog. Regressions lines are drawn with a confidence interval of 0.95 (highlighted using translucent bands around the regression line).

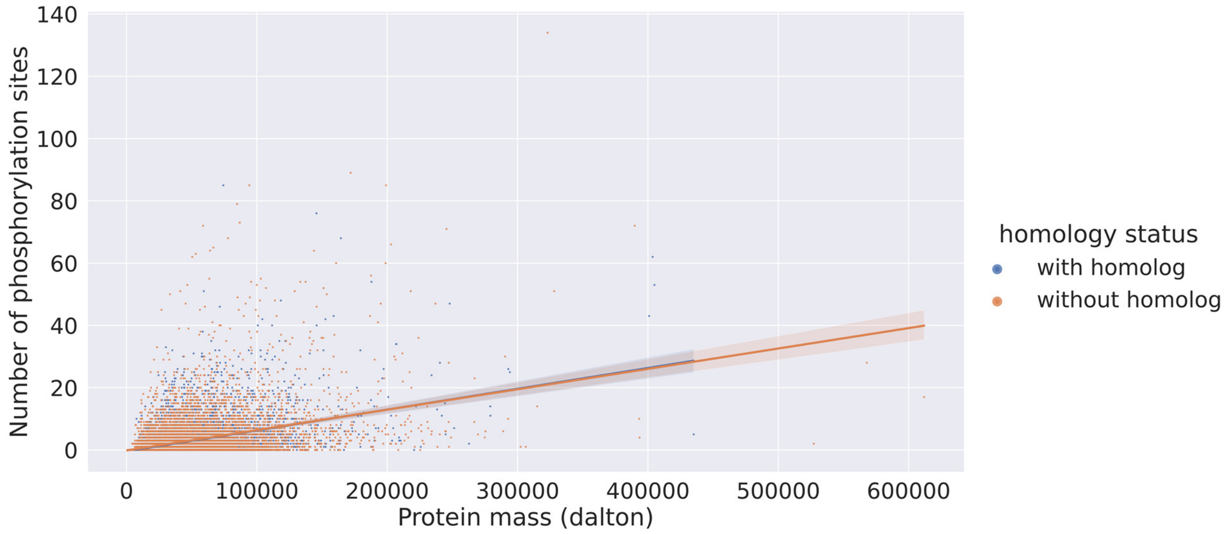

Figure S8 Plot of the relationship between masses and the number of phosphorylation sites for each phosphorylated protein in Arabidopsis thaliana

Data in blue are for proteins that have at least one homolog in another species while data in orange are for proteins without homolog. Regressions lines are drawn with a confidence interval of 0.95 (highlighted using translucent bands around the regression line).
