# Supplementary material for: Evolutionary Divergence of Phosphorylation to Regulate Interactive Protein Networks in Lower and Higher Species
Source: Int J Mol Sci. 2022 Nov 20;23(22):14429. doi: 10.3390/ijms232214429 (PMC9697241; doi:10.3390/ijms232214429)
Supplement: Supplementary file 1 [file ijms-23-14429-s001.zip › supplementary_tables.pdf]

Supplementary tables

Table S1. Enrichments with GO Biological Process for human protein deciles built according to the number of phosphorylation sites in proteins For each decile, the significant annotations (which are associated with an adjusted p-value < 0.05) are listed on a table in the Excel sheet. In each tab, the annotations obtained for the other deciles are also displayed. The last tab groups, for all deciles, the annotations that have been identified with a significant threshold for at least one decile.

Table S2. Selected GO enrichments identified for *H. sapiens*, *D. melanogaster* and *S. cerevisiae* proteins grouped according to their number of phosphorylation sites

Table S3. Enrichments with GO Biological Process for *D. melanogaster* proteins grouped according to the number of phosphorylation sites in proteins For each bin, the significant annotations (which are associated with an adjusted p-value < 0.05) are listed on a table in the Excel sheet. In each tab, the annotations obtained for the other bins are also displayed. The last tab groups the annotations that have been identified with a significant threshold for at least one bin.

Table S4. Enrichments with GO Biological Process for *S. cerevisiae* proteins grouped according to the number of phosphorylation sites in proteins For each bin, the significant annotations (which are associated with an adjusted p-value < 0.05) are listed on a table in the Excel sheet. In each tab, the annotations obtained for the other bins are also displayed. The last tab groups the annotations that have been identified with a significant threshold for at least one bin.

Table S5. List of 9 kinases group with the associated number of phosphorylated proteins

Table S6. Representative annotations obtained by enrichment analysis of the protein lists corresponding to the different clusters in Figure 10

Table S7. Summary of the number of phosphorylation variation per protein reported for 6 time point after EGF exposure.

Tables S8-S13. Modules of interacting proteins differentially phosphorylated at 1mn, 5mn, 10mn, 15mn, 20mn and 30mn after EGF exposure The sites were identified with AMINE (Active Module Identification through Network Embedding – Pasquier et al. 2021).

Table S14. Enrichments with annotations from Gene Ontology (Biological Process), KEGG, Reactome and WikiPathway of the most striking module identified with AMINE at 1mn, 5mn, 10mn, 15mn, 20mn and 30mn after EGF exposure
